# Ab-interno surgical technique for the implantation of a wireless subretinal prosthesis in mini-pigs

**DOI:** 10.1038/s41598-020-75579-4

**Published:** 2020-10-28

**Authors:** Kwang-Eon Choi, Vu Thi Que Anh, Hee Won Seo, Namju Kim, Sohee Kim, Seong-Woo Kim

**Affiliations:** 1 Department of Ophthalmology, Korea University College of Medicine, Seoul, South Korea; 2 Department of Ophthalmology, Hanoi Medical University, Hanoi, Vietnam; 3 Department of Robotics Engineering, Daegu Gyeongbuk Institute of Science and Technology (DGIST), Daegu, South Korea

**Keywords:** Hereditary eye disease, Macular degeneration, Electrical and electronic engineering, Visual system

## Abstract

We sought to describe the surgical techniques required in the ab-interno method to implant subretinal prostheses in mini-pigs and suggest tips to facilitate optimal outcomes. During vitrectomy, the use of valved trocar cannulas was essential to stabilize the detached retina and implanted chip. As a first step in retinal detachment, a 23-gauge cannula with very small amount of viscoelastic material was used to establish the retinal hole and promote retinal detachment. Then, balanced salt solution was applied to increase the retinal detachment and diathermy was used to make opening for subretinal prosthesis. For easy positioning of the subretinal prosthesis, a curved laser probe was adopted when handling the subretinal prosthesis under the retina. After surgery, the sclerotomy sites were tightly sutured to prevent silicone oil leakage. Without special equipment, such as a 41-gauge tip, retinal detachment could be induced easily, while the prosthesis was also successfully inserted and manipulated under the retina without an iatrogenic retinal tear. Two weeks after the operation, the oil fully occupied the intraocular volume without leakage. The subretinal prosthesis remained stable without complication. Understanding the principle of the ab-interno method and considering several tips for improving surgical access may help to enhance surgical success rates of subretinal prostheses implantation.

## Introduction

In degenerative retinas, such as those with retinitis pigmentosa or dry age-related macular degeneration, visual loss is the result of damage to photoreceptors. In advanced stages of such retinal degeneration, surgical implantation of retinal prostheses has been the foremost commercially available treatment option^1–4^. Each device requires a different surgical approach for implantation because of its design and intended location^5–10^. Recently, PRIMA implant has been introduced to the market as a wireless subretinal micro photovoltaic chip and can be implanted via an ab-interno approach^11–17^. Considering interim clinical trial results from Europe and relative ease of the surgery^18^, development and implantation of this type of retinal prosthesis is likely to rapidly increase the need for surgeons to perform the surgery more safely and comfortably^19–29^. However, current methods for subretinal surgery as well as ab-interno subretinal implantation have not been widely taught to current-generation retinal surgeons, with subretinal surgical approaches being more widely discussed and published on in the late 1990s and early 2000s^30,31^. Although ab-interno surgery for subretinal implantation is easier than ab-externo surgery^24,32^, this surgical procedure still requires an experienced operator and involves the conduct of inconvenient procedures using additional instruments, such as connecting a 41-gauge cannula to the vitrectomy machine.

Here, we presented an ab-interno method for the induction of retinal detachment and manipulation of the subretinal prosthesis and offer several tips focused on reducing surgical difficulties.

## Results

### Surgical technique and short-term results

During vitrectomy, valved trocar cannulas were used to stabilize the intraocular volume^33^. Three-port, 23-gauge vitrectomy (Associate; Dutch Ophthalmic Research Center B.V., Zuidland, the Netherlands) was performed with an indirect BIOM lens (Oculus Biom Ready; Oculus Surgical, Inc., Port St. Lucie, FL, USA). Three ports were prepared by inserting trocar cannulas into the sclera at 3 mm from the limbus on the ventromedial, ventrolateral, and dorsomedial sides, respectively. The vitreous was removed using a vitreous cutter while continually supplying balanced salt solution (BSS) (Alcon, Fort Worth, TX, USA). Anterior capsule-saving lensectomy was also performed.

**Figure 1:**
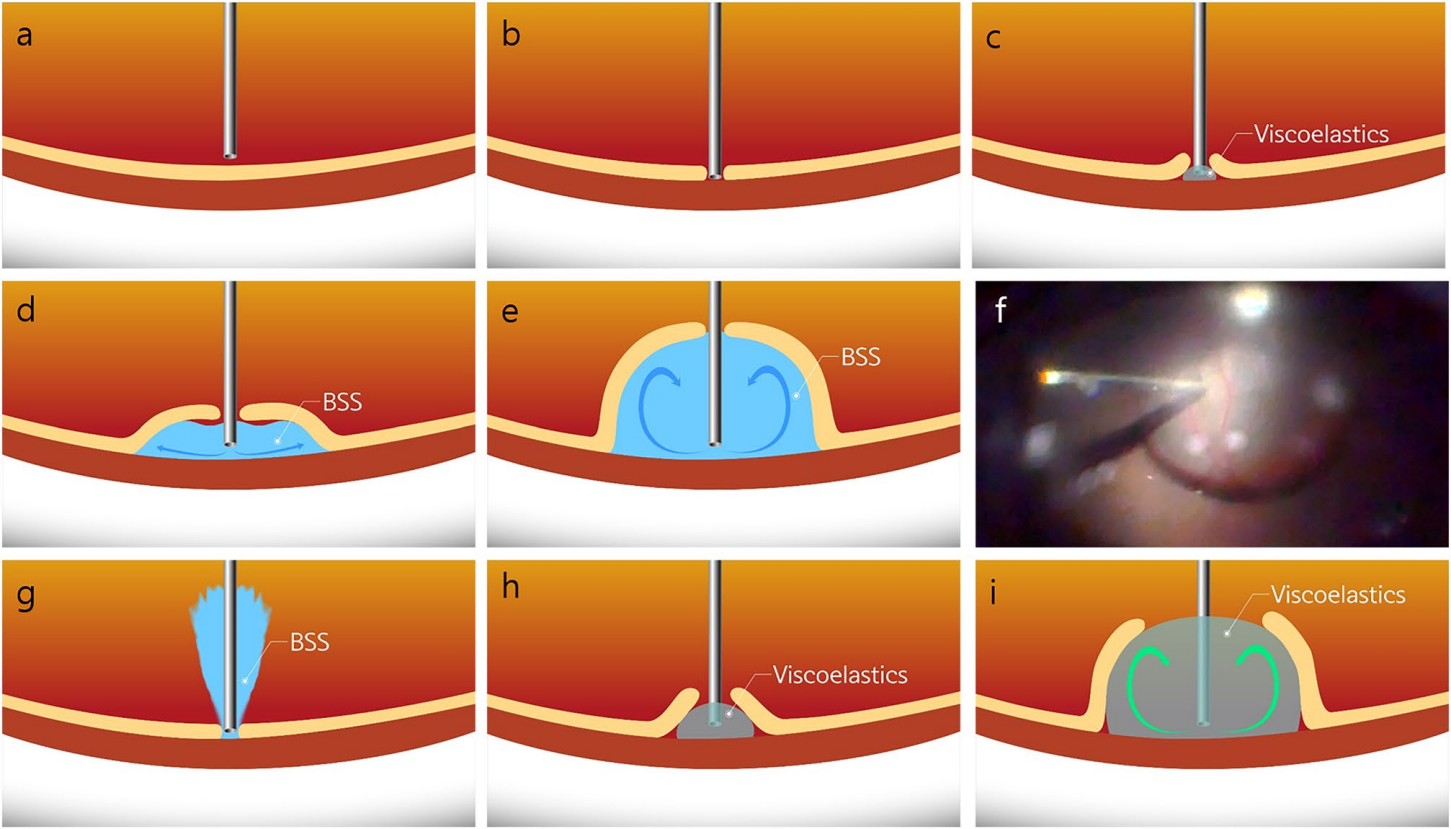
Schematic images of the initiation and enlargement of the subretinal detachment using various methods. **(a,b)** A 23-gauge cannula was used to approximate the retina. **(c)** Focal retinal detachment was obtained by injecting less than 0.1 cc of viscoelastic material. **(d–f)** After focal detachment was achieved using viscoelastic materials, BSS was injected to fill the subretinal area. **(g)** Focal retinal detachment was not well-induced due to the backflow of BSS. **(h,i)** In the process of widening the subretinal detachment, the initial hole inadvertently tore into a large tear when only viscoelastic materials were used to create the retinal detachment.

**Figure 2:**
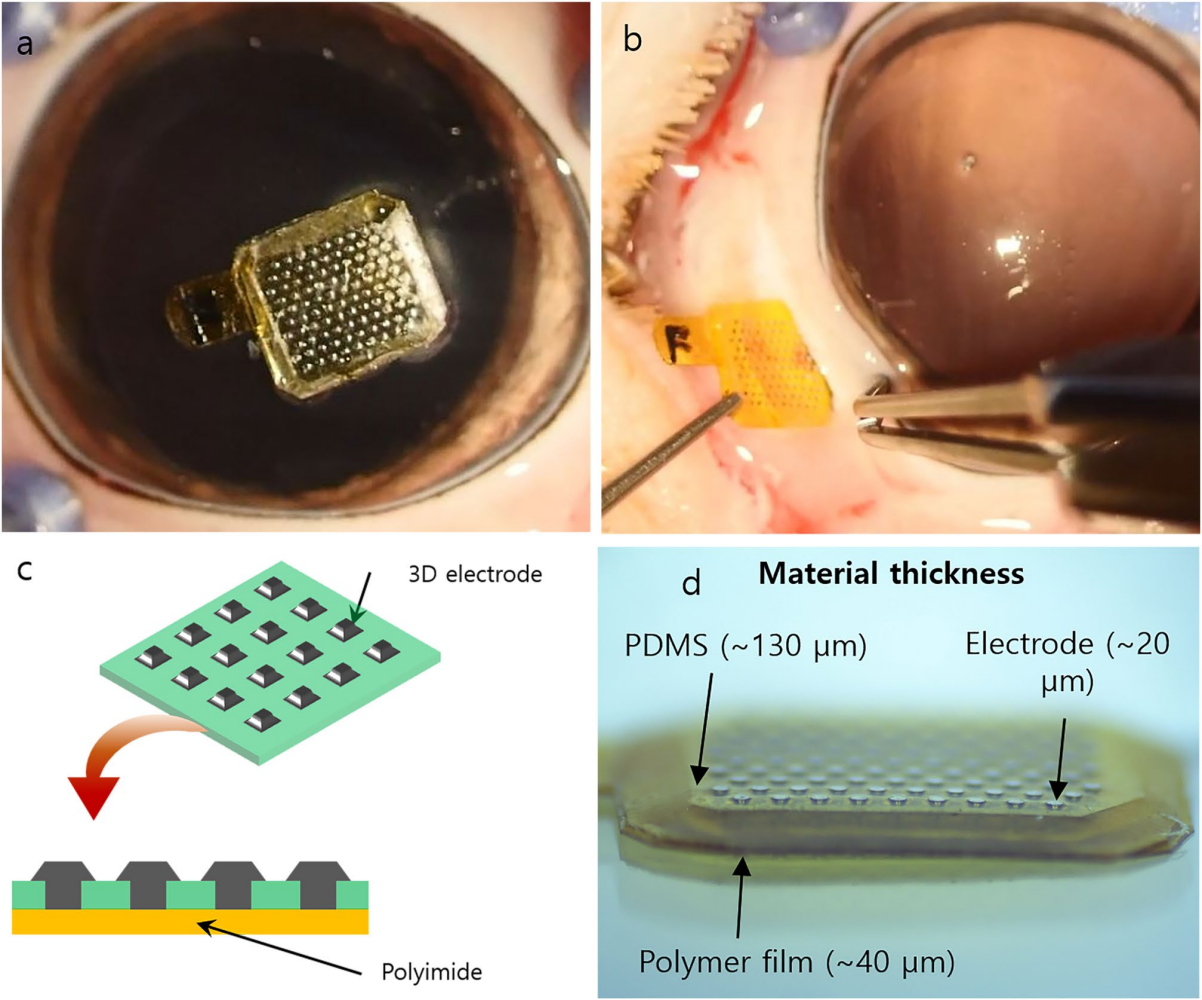
Subretinal prosthesis and insertion.** (a)** The overall size of the subretinal prosthesis was 4.5 mm × 5 mm. **(b)** Through a scleral incision of about 4.5 mm in length, a retinal prosthesis was inserted into the vitreous cavity. **(c,d)** Schematic structures of the subretinal prosthesis.

A small hole was made by pressing the retina lightly with a 23-gauge cannula of viscoelastic material at the superonasal peripheral retina, and less than 0.1 cc of viscoelastic material was injected into the retinal hole to induce a tiny focal retinal detachment. Next, the cannula with BSS was inserted into the subretinal cavity and the BSS was gently injected to increase the size of the retinal detachment (Fig. 1) (see Supplementary Video S1 and S2 online). Once the retinal detachment was large enough that it included the peripheral retina, a scleral incision was made with a 2.75-mm slit knife at 1.5 mm from the limbus of the dorsolateral or dorsal side. Next, an incision approximately measuring 5 mm in length was completed (Fig. 2).

**Figure 3:**
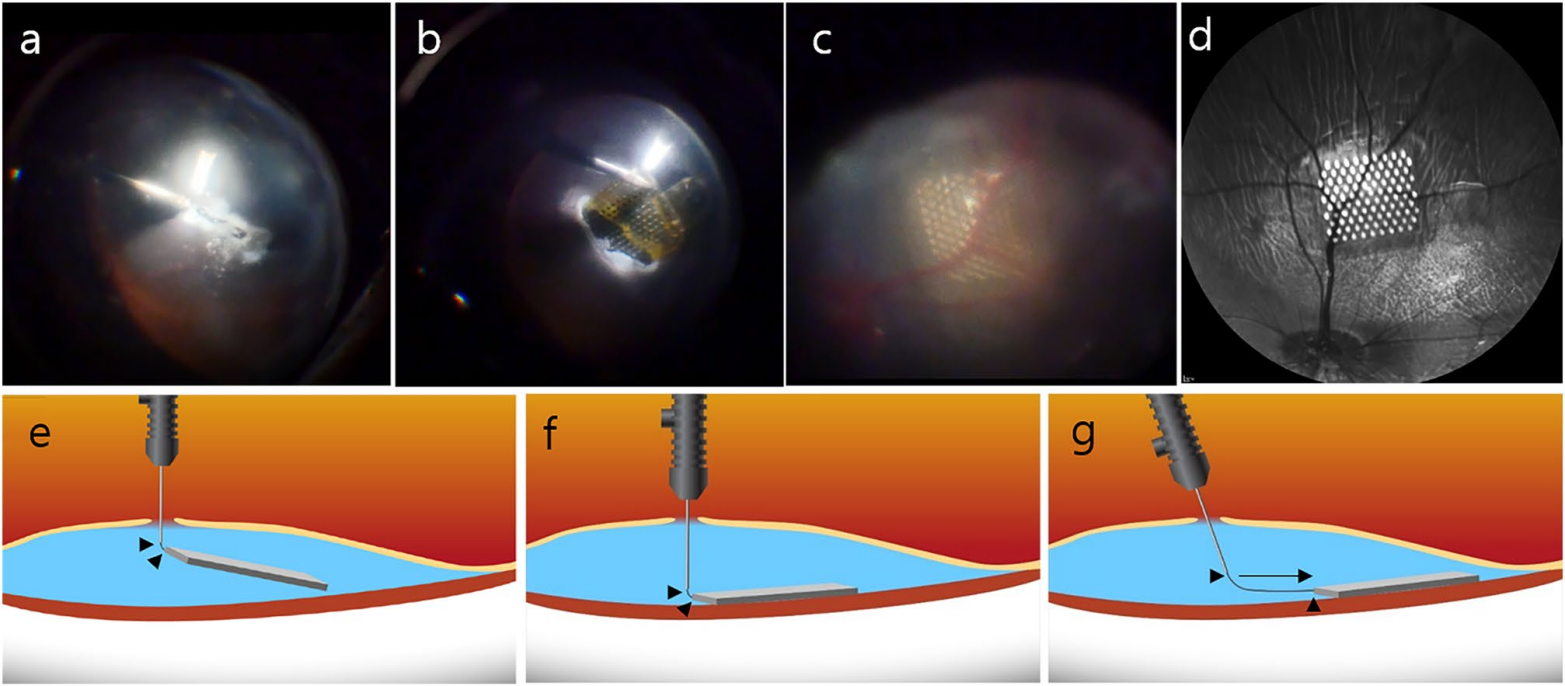
Enlargement of the retinal hole and insertion of the subretinal prosthesis. **(a)** Diathermy was used and the hole was gradually widened by about 4.5 mm. **(b)** The subretinal prosthesis was inserted into the subretinal space with micro-forceps. **(c)** A curved directional laser probe tip was used to push the implanted prosthesis forward and adjust its position. **(d)** After the oil tamponade, the subretinal prosthesis was settled into its desired location. **(e–g)** Schematic images of manipulation of the plate using a curved laser probe in the subretinal space. A directional laser probe with a fixed fiber and movable shaft measuring 3.2 mm in height to its tip and 0.54 mm wide at the tip was used. **(e,g)** Press down the subretinal prosthesis with the laser probe without projection of curved fiber (black arrowhead). **(f,g)** After insertion of the subretinal prosthesis, the curved fiber (black arrowhead) of the laser probe was directed away (black arrow) from the actuation button in the subretinal space.

Diathermy was used to create a hole to insert the subretinal prosthesis under the retina and the hole was gradually widened by about 4.5 mm (see Supplementary Video S1 online). The retinal prosthesis was inserted into the subretinal space with micro-forceps (Fig. 3). During the insertion of the retinal prosthesis under the retina, the partial air-fluid exchange was performed to decrease the height of retinal detachment for preventing retinal prosthesis from turning over. To mitigate the possibility of an iatrogenic retinal tear, a curved directional laser probe tip (23-gauge directional endo ocular laser probe; synergetics USA, Inc., O'Fallon, MO, USA) with an expandable fiber or a moving shaft was applied to push the implanted prosthesis forward and adjust its position (Fig. 3) (see Supplementary Video S3 and S4 online). Under the detached retina, the retinal prosthesis could be driven into the visual streak with the force of inertia created by shaking the eye back and forth (see Supplementary Video S5 online). After confirming that the subretinal prosthesis was located in the desired position, air-fluid exchange was performed. Endolaser photocoagulation was carried out around the retinotomy site and oil tamponade was completed. All port sites were sutured with 9-0 Prolene (Johnson & Johnson, New Brunswick, NJ, USA) to prevent postoperative oil leakage.

**Figure 4:**
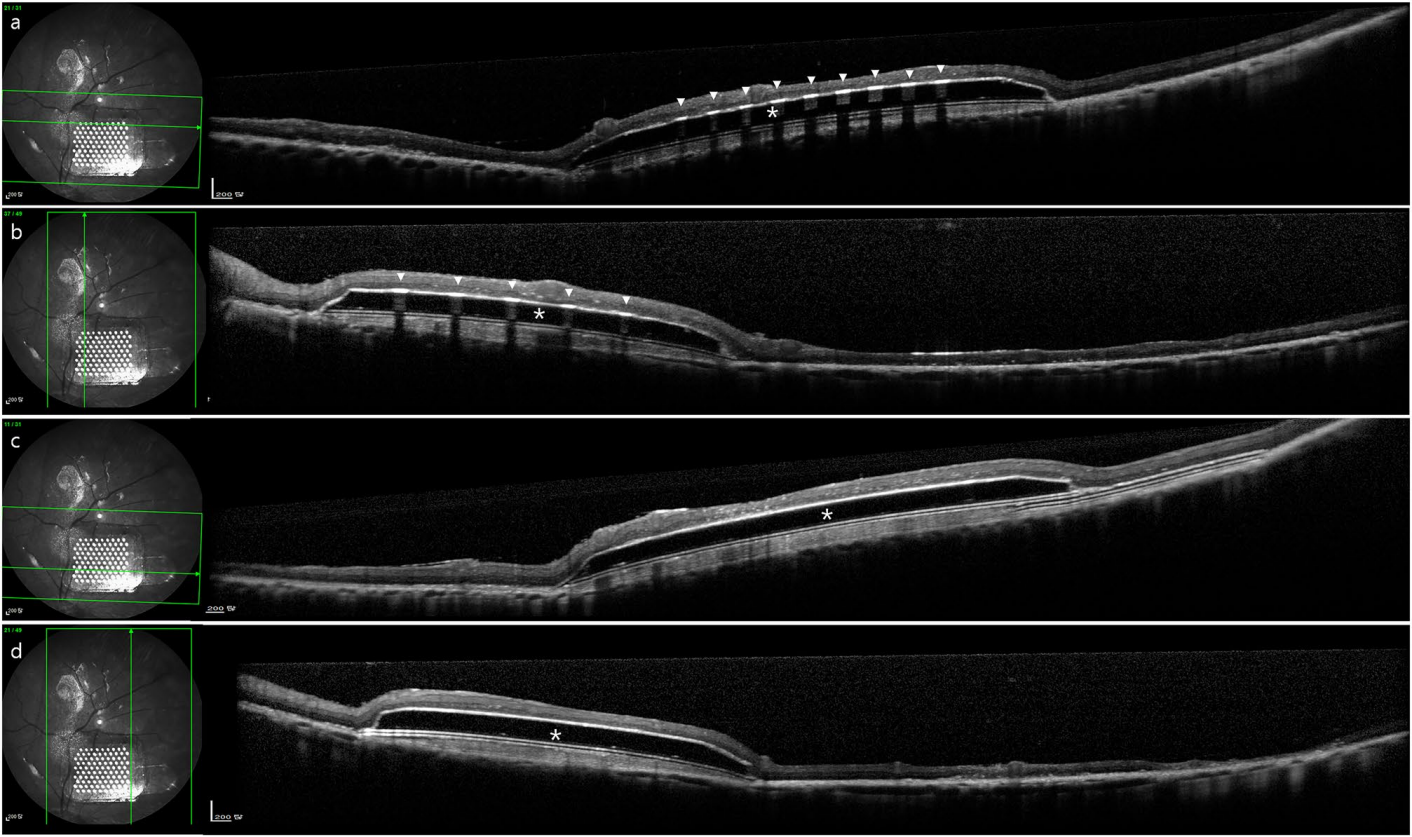
Optical coherence tomography results obtained two weeks after subretinal implantation in both successful and failed cases. **(a,b)** Vertical and horizontal views of optical coherence tomography images indicate stable status on the three-dimensional protruded electrodes (white arrowheads) of the subretinal prosthesis. **(c,d)** Vertical and horizontal views of optical coherence tomography images indicate stable status on the base (white asterisk) of the subretinal prosthesis.

The mean age of the four pigs was 9.75 ± 0.96 months and their mean axial length was 20.49 ± 0.71 mm. Two weeks after the operation, the retinal prostheses were well maintained in the subretinal space without proliferative vitreoretinopathy or retinal detachment. On optical coherence tomography, the subretinal prostheses were observed to be well located under the retina (Fig. 4).

## Discussion

There has been an ab-interno surgical method using a protocol of inducing retinal detachment by first injecting BSS and then continuously infusing viscoelastic material into the subretinal space. When BSS is injected manually with a 23-gauge cannula, making retinal detachment without the viscoelastic material is possible in some cases (see Supplementary Video S6 online). However, when the retinal hole is large, it is difficult to initiate and continue focal retinal detachment due to the backward flow of the BSS into the vitreous cavity (Fig. 1) (see Supplementary Video S7 online). For this reason, a machine-driven fluid-stream injection approach with a 41-gauge cannula was successfully adopted instead^32^. This method is useful but requires a 41-gauge cannula connected to the silicone oil infusion pump of a vitrectomy machine to support a controllable, steady, and stable procedure. The procedure to connect a BSS-filled 41-gauge cannula to the vitrectomy machine and exchange the 41-gauge cannula again with a silicone oil syringe during the surgery is quite inconvenient for the surgeon to complete. Thus, we induced retinal detachment by slightly pressing with a 23-gauge cannula to create a retinal hole and subsequently injected a small amount of viscoelastic material manually to produce a tiny subretinal space that would allow the BSS to spread subretinally (see Supplementary Video S1 and S2 online). The initial small retinotomy site could inadvertently become a much larger tear if we continued to inject viscoelastic material into the subretinal space to increase the retinal detachment size (Fig. 1) (see Supplementary Video S8 and S9 online). In addition, injecting the full amount of viscoelastic material into the subretinal space would limit manipulation of the subretinal prosthesis because the implant would be floating in space somewhere between the retina and the retinal pigment epithelial layer. In addition, significant time and effort are required to remove the viscoelastic material after positioning the subretinal prosthesis. During removal of the viscoelastic material, the retinotomy can become enlarged further and inadvertent retinal tissue tear by the cutter or aspiration tip is common. Finally, the implanted subretinal prosthesis may inevitably move backward toward the retinotomy site or even exit into the vitreous cavity in conjunction with the removal of the viscoelastic material. To avoid such difficulties, injecting a large amount of BSS into the subretinal space with or without small amount of the viscoelastic material is an adequate mean to facilitate the insertion and positioning of a wireless retinal in the desired position (Fig. 1) (see Supplementary Video S1 and S6 online).

Another problem that may be encountered after successful insertion of the subretinal prosthesis involves positioning. Although micro-forceps can be used to manipulate a subretinal prosthesis under the retina, retinal tears often become unintentionally larger along the shaft of the micro-forceps (see Supplementary Video S10 and S11 online). Instead, it is preferable to move the subretinal prosthesis with a curved rod; either a directional curved laser probe with an expandable fiber or a moving shaft can be used for this procedure. A curved laser probe allows the degree of curve tip length to be adjusted by the operator with a button on the handle, enabling more minute manipulation of the subretinal prosthesis without contact between the shaft of the instrument and retina (Fig. 3) (see Supplementary Video S3 online).

The abrupt uncontrolled fluctuation of eyeball volume during surgery is likely to cause unintended intraretinal damage such as an iatrogenic retinal tear and choroidal detachment. In this regard, we recommend using a valved-trocar system to minimize fluid flow inside the eye. Pigs and humans have different corneal and scleral thicknesses and scleral elasticity profiles^34–36^. It is important to suture every sclerotomy site, although we used a sutureless vitrectomy system in our pig experiment. The amount of oil leakage through the sclerotomy sites during or after surgery was much larger than what we expected in this study and postoperative retinal re-detachment was observed in one case of sutureless oil tamponade.

This study has some limitations. First, we could not show long-term postoperative stability and statistical significance in a large number of animals. Instead, we aimed to describe the surgical methods of ongoing experiments in the present study. We plan to present the long-term outcomes of subretinal prostheses placed using this surgical procedure in mini-pigs in a future article. Second, this surgical procedure was performed in normal pigs. Surgery in the degenerated retina is much more difficult than that in the healthy retina because the remaining inner retina is very atrophic or scar tissue is abnormally adhesive to subretinal tissues. A study on the implantation of retinal prostheses in an outer retinal degeneration animal model is being conducted at this time.

It is essential to improve the relevant surgical techniques to facilitate better outcomes in the implantation of retinal prostheses. The detailed refined ab-interno approach may constitute a means by which to achieve successful wireless retinal prosthesis implantation.

## Materials and methods

### Animals

Mini-pigs (Micropig; Apures Co., Ltd., Pyeongtaek-si, Korea) were placed under general anesthesia by the intravenous injection of alfaxalone (Alfaxan 1 mg/kg; Vetoquinol, Lure, France) into the marginal auricular vein following premedication with a subcutaneous injection of atropine (0.05 mg/kg) and an intramuscular injection of xylazine (Rompun 1 mg/kg; Bayer Corp., Pittsburg, PA, USA) and azaperone (4 mg/kg). Following the induction of general anesthesia, each eye was irrigated with 5% povidone iodine and draped for surgery.

All procedures adhered to the Association for Research in Vision and Ophthalmology (ARVO) Statement for the Use of Animals in Ophthalmic and Vision Research (ARVO Animal Policy). Approval for this study was obtained from the Institutional Animal Care and Use Committee of Korea University’s College of Medicine.

### Subretinal prosthesis

The overall thicknesses of three-dimensional electrodes were approximately 196 μm. The three-dimensional electrodes had the base thickness of 130 μm and protrusions with a height of about 20 μm. The materials used were silicon for the electrode sites and polydimethylsiloxane (PDMS) for the transparent base of the electrodes. The detailed fabrication procedures are described elsewhere^37^. These electrodes were combined with a 40-μm-thick polyimide film, using glue. The combined chip was coated with 3-μm-thick parylene-C to assure the biocompatibility. The overall chip size was 4.5 mm × 5 mm (Fig. 2).

## Supplementary information

Supplementary Video 1.

Supplementary Video 2.

Supplementary Video 3.

Supplementary Video 4.

Supplementary Video 5.

Supplementary Video 6.

Supplementary Video 7.

Supplementary Video 8.

Supplementary Video 9.

Supplementary Video 10.

Supplementary Video 11.

## Data Availability

The datasets generated during and/or analyzed during the current study are available from the corresponding authors on reasonable request.
